# Prognostic model for long-term survival of locally advanced non-small-cell lung cancer patients after neoadjuvant radiochemotherapy and resection integrating clinical and histopathologic factors

**DOI:** 10.1186/s12885-015-1389-4

**Published:** 2015-05-06

**Authors:** Christoph Pöttgen, Martin Stuschke, Britta Graupner, Dirk Theegarten, Thomas Gauler, Verena Jendrossek, Lutz Freitag, Jehad Abu Jawad, Eleni Gkika, Jeremias Wohlschlaeger, Stefan Welter, Matthias Hoiczyk, Martin Schuler, Georgios Stamatis, Wilfried Eberhardt

**Affiliations:** 1 Department of Radiotherapy, West German Cancer Center, University of Duisburg-Essen, Essen, Germany; 2 Institute of Pathology and Neuropathology, West German Cancer Center, University of Duisburg-Essen, Essen, Germany; 3 Department of Medical Oncology, West German Cancer Center, University of Duisburg-Essen, Essen, Germany; 4 Institute of Cell Biology (Cancer Research), University of Duisburg-Essen, Essen, Germany; 5 Division of Interventional Pneumology, Ruhrlandklinik,West German Lung Center, University of Duisburg-Essen, Essen, Germany; 6 Division of Thoracic Surgery and Thoracic Endoscopy, Ruhrlandklinik, West German Lung Center, University of Duisburg-Essen, Essen, Germany; 7 Division of Thoracic Oncology, Ruhrlandklinik, West German Lung Center, University of Duisburg-Essen, Essen, Germany; 8 German Cancer Consortium (DKTK), Heidelberg, Germany

**Keywords:** Prognostic model, Long-term survival, NSCLC, Histopathological response

## Abstract

**Background:**

Outcome of consecutive patients with locally advanced non-small cell lung cancer and histopathologically proven mediastional lymph node metastases treated with induction chemotherapy, neoadjuvant radiochemotherapy and thoracotomy at the West German Cancer Center between 08/2000 and 06/2012 was analysed. A clinico-pathological prognostic model for survival was built including partial or complete response according to computed tomography imaging (CT) as clinical parameters as well as pathologic complete remission (pCR) and mediastinal nodal clearance (MNC) as histopathologic factors.

**Methods:**

Proportional hazard analysis (PHA) and recursive partitioning analysis (RPA) were used to identify prognostic factors for survival. Long-term survival was defined as survival ≥ 36 months.

**Results:**

A total of 157 patients were treated, median follow-up was 97 months. Among these patients, pCR and MNC were observed in 41 and 85 patients, respectively. Overall survival was 56 ± 4% and 36 ± 4% at 24 and 60 months, respectively. Sensitivities of pCR and MNC to detect long-term survivors were 38% and 61%, specificities were 84% and 52%, respectively.

Multivariable survival analysis revealed pCR, cN3 category, and gender, as prognostic factors at a level of α < 0.05. Considering only preoperative available parameters, CT response became significant. Classifying patients with a predicted hazard above the median as high risk group and the remaining as low risk patients yielded better separation of the survival curves by the inclusion of histopathologic factors than by preoperative factors alone (p < 0.0001, log rank test). Using RPA, pCR was identified as the top prognostic factor above clinical factors (p = 0.0006). No long term survivors were observed in patients with cT3-4 cN3 tumors without pCR.

**Conclusions:**

pCR is the dominant histopathologic response parameter and improves prognostic classifiers, based on clinical parameters. The validated prognostic model can be used to estimate individual prognosis and forms a basis for patient selection for treatment intensification.

## Background

Mediastinal nodal clearance (MNC) defined as sterilization of initially involved (cN2, cN3) mediastinal lymph nodes after neoadjuvant radiochemotherapy followed by surgery (ypN0) was identified as a prognostic marker for long-term survival for stage III non-small-cell lung cancer (NSCLC) [1-5]. In these trials, the proportion of patients with MNC among patients who received thoracotomies after neoadjuvant radiochemotherapy was approximately 50%. MNC was taken as a surrogate endpoint for the treatment effect of neoadjuvant radiochemotherapy and represented the primary endpoint of the Radiation Therapy Oncology Group phase II trial 02–29 [6]. In this trial, full dose radiotherapy to 61.2 Gy in conventional fractionation and weekly concurrent carboplatin/paclitaxel chemotherapy was given. Nodal clearance was achieved in 63% of patients with pathologically proven N2 or N3 involvement receiving thoracotomy, but only 8% had a pathologic complete remission (pCR), defined as sterilization of tumor tissue at all involved sites (primary and lymph nodes). Generally, pCR is found at lower frequencies than MNC in patients undergoing thoracotomy after neoadjuvant radiochemotherapy and has been observed in 8-39% of patients in larger series [1,2,5-10]. Others found that pCR is an important prognostic factor for survival after neoadjuvant radiochemotherapy and resection [9-11].

In this large retrospective monoinstitutional study, we evaluated the prognostic strengths of patient characteristics, treatment variables and histopathologic response parameters within a prognostic model for survival in a comprehensive group of patients with locally advanced NSCLC and histopathologically proven mediastinal lymph node metastases undergoing neoadjuvant radiochemotherapy and thoracotomy. In particular, the prognostic values of pCR and MNC on long-term survival were analyzed.

## Methods

This observational study included data from consecutive patients with stage IIIA/IIIB NSCLC according to the UICC classification, 7th edition 2009, who were treated at the West German Cancer Center between August 2000 and June 2012.

The study has been approved by the Ethics committee of the University of Duisburg-Essen, Essen, Germany. All treatments were performed in accordance with the German Legislation of Radiation Protection. Written informed consent was obtained from all patients prior to treatment initiation.

Following induction chemotherapy and neoadjuvant chemoradiotherapy, all patients were resected at the Division of Thoracic Surgery, Ruhrlandklinik, Essen. All patients were staged by mediastinoscopy or endobronchial ultrasound-guided needle aspiration to confirm cN2 or cN3 status by histo- or cytopathology. The additional workup included computed tomography (CT) scans of the thorax and abdomen and bone scans, or whole body PET/CT which became available at our institution in 2002. In addition, CT or MRI scans of the brain were performed in all patients. All patients were discussed in the multidisciplinary tumor board of the lung cancer center of the West German Cancer Center. Following three cycles of induction chemotherapy (cisplatin/paclitaxel doublets predominantly given throughout the whole time period, cisplatin/etoposide was administered to patients prior to 2007) concurrent radiochemotherapy was initiated [7]. Cisplatinum-based doublets were used during the concurrent chemoradiotherapy phase. Before 2004, etoposide was predominantly applied which was later replaced by vinorelbine as combination partner for cisplatin. Patients received conventionally fractionated radiotherapy to 44–46 Gy at 2 Gy per fraction, or accelerated hyperfractionated radiotherapy at 2 × 1. 5 Gy per day, ≥6 h interval, 5 days a week, up to a total dose of 45 Gy. Three-dimensional treatment planning was performed for all patients based on planning CT scans following induction chemotherapy. The planning procedure has been previously described in detail [7].

During the last week of neoadjuvant radiochemotherapy, patients underwent restaging CT-scans and were again discussed in the multidisciplinary tumor board. Preoperatively, re-mediastinoscopy was performed in all patients with initial N3-disease to define the response to induction treatment. Patients with positive contralateral nodes at repeated mediastinoscopy were excluded from surgery and were offered definitive concurrent radiochemotherapy. Those patients who were evaluated as resectable by the thoracic surgeon at that time point were offered surgery. Criteria of resectability have been described earlier [12]. Patients underwent a preoperative cardiovascular risk assessment including a cardiopulmonary function test. Patients were ineligible due to a predicted postoperative forced expiratory volume at 1 s of less than 1 liter (quantitative ventilation–perfusion lung scanning), cardiac infarction or unstable angina pectoris during the 6 months before study entry or cardiac failure of class III or greater (NYHA criteria). If re-evaluation showed continuing medical/functional and technical operability, patients were taken to thoracotomy 3–5 weeks after the end of radiation treatment.

After treatment completion, no adjuvant treatment was planned and patients were monitored every three months during the first year of follow-up. Thereafter, examination intervals were set to 4–6 months. Time to progression (TTP) and overall survival (OS) were calculated from the start of the induction chemotherapy. For non-progressing patients, TTP was calculated as censored at the time of the last follow-up visit. Survival information was updated from the local residents registration offices between 1st and 15^th^ of September 2013. Toxicities were assessed using CTC (v. 2) scores [13].

Pathologic complete response (pCR) was defined as complete disappearance of vital tumor cells at all initially involved tumor sites (ypT0 ypN0) assessed by histopathologic examination of the resection specimen. Regression grades 2A and 2B were defined as evidence of therapy-induced tumor regression with >10% and <10% of vital tumor cells remaining, respectively [14]. MNC was defined as ypN0. Histopathologic complete regression at the primary tumor site only (pCR-T) was defined as ypT0.

Long-term survivors were defined as patients living ≥36 months since start of induction chemotherapy. Twenty percent of all deceased patients (n = 98) of this cohort fulfilled the criteria for long-term survivors. On the other hand, 47% of the living patients had a follow-up time of less than 36 months. For these patients, the conditional probability p was calculated to survive 3 years having the observed survival time according to a backward selection proportional hazard model. These patients were included in the analysis with weight p as long-term survivors and with weight 1-p as short-term survivors, respectively.

Sensitivities and specificities of the histopathologic regression parameters to predict long-term survival as well as the positive likelihood ratio = sensitivity/(1-specificity) together with its 95% confidence limits and the Cochrane-Mantel-Haenszel (CMH) test for the association of the histopathologic response parameters with survival were calculated with the procedure FREQ using SAS statistical software version 9.2 (Cary, NC). Proportional hazard regression analysis (PHA) was performed with the procedure PHREG. The following clinical explanatory variables were included in the full proportional hazard regression model. Patient-dependent variables: age as a continuous variable, gender, Charlson comorbidity score. Tumor characteristics: cN2 (yes vs. no), cN3, Pancoast tumor localisation, cT3, cT4, stage IIB, adenocarcinoma (yes vs. no), squamous cell carcinoma (yes vs. no), grade 3 carcinoma, number of histopathologically proven mediastinal lymph node metastases at initial staging. Treatment characteristics: hyperfractionated accelerated radiotherapy (yes vs. no), cisplatin/paclitaxel doublet as induction chemotherapy, cisplatin/vinorelbine as concurrent chemotherapy, pneumonectomy, R1 resection, R2 resection. In addition, the following histopathologic and clinical response variables were included: pCR, MNC, regression grade (regression grade ≥ 2B, regression grade > 2A, ypT0), response to neoadjuvant radiochemotherapy according to computed tomography imaging studies before and after neoadjuvant radiochemotherapy according to the RECIST criteria (PD/NC versus PR/CR) [15]. A backward variable elimination procedure was used to retain variables in the model significant at α = 0.05. The proportional hazard assumption was studied by Schoenfeld partial residuals and their correlation with the rank order of failure times and by introducing time dependent interaction terms for the covariates in the model to detect a possible trend over time of the hazard ratio [16,17].

Prognostic models were derived using the PHA variable estimates after backward elimination with or without inclusion of the histopathologic parameters. The sample was split in equally sized high risk or low risk groups depending on whether the prognostic index, defined as the vector product of the PHA regression coefficients and the patients’ expression of prognostic parameters constituting a factor of the hazard function, was located above or below the median, respectively. Ten-fold cross-validation was used to evaluate the predictive accuracy of the prognostic model on a data set independent of the one used for model building. Therefore, the whole sample of 157 patients was split into 10 approximately equally sized subgroups by random numbers. For each k-th subgroup, the prognostic model was developed from scratch on the basis of the data from the 9 other subgroups by fitting the PHA model with backward elimination. This model was then used to split the k-th subgroup into a high risk and low risk group. Not necessarily the same variables had to stay in the model for each of the k loops of cross-validation. Repeating this process for all subgroups resulted in a split of the whole sample into a high and a low risk group. The respective Kaplan-Meier curves were termed as cross-validated Kaplan Meier curves [18]. The prognostic model containing clinical and histopathological covariables was evaluated against the standard model using the difference of the log-rank statistics for comparison of the cross-validated Kaplan Meier curves of the high and low risk groups according to both models as a test statistic [18].

For regression tree growing during recursive partitioning analysis (RPA), the log rank test was used as the splitting criterion [19]. The dichotomic prognostic parameter that results in the largest separation of 5 year survival at each node with a p-value < 0.05 was selected as the criterion for this node.

## Results

**Figure 1:**
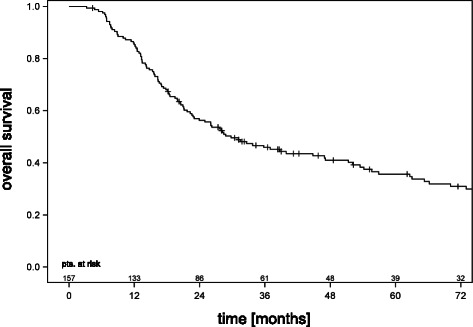
Survival curve for all patients of this study.

Median follow-up for survival of the entire cohort was 97 months. Patient characteristics have been reported in a previous publication on the effects of accelerated hyperfractionation on pCR and are summarized in Table 1 [7]. One hundred fifty-seven patients have been resected out of 164 who underwent the induction chemoradiotherapy phase completely. Eight patients were not resected either due to comorbidities (n = 3), persistent contralateral lymph node involvement at remediastinoscopy (n = 3), or patients’ refusal (n = 2), and received definitive radiochemotherapy (n = 5), or palliative treatment (n = 3), respectively. Pneumonectomies were performed in 40 patients. In this cohort, none of the patients died within 30 days after surgery. Figure 1 shows the survival curve of all patients who underwent tumor resection (n = 157). Two-, three-, and five- year survival was 56 ± 4%, 46 ± 4%, and 36 ± 4%, respectively.

**Table 1:** **Patient characteristics**

	No.	*[%]*
Total	157	*100*
**Sex**		
**Male/Female**	94/63	*60/40*
**Age, years**		
median (range)	59 · 0 (34–74)	
**Primary tumor and lymph node classification**		
cT1-3 N2	81	*52*
cT1-3 N3	35	*22*
cT4 N2	37	*24*
cT4 N3	4	*2*
**Histology**		
Squamous Cell Carcinoma	58	*37*
Adenocarcinoma	75	*48*
Large Cell Carcinoma	16	*10*
not differentiated, NOS	7	*4*
sarcomatoid	1	*1*
**G**		
1	3	*2*
2	50	*32*
3	94	*60*
x	10	*6*
**Localisation**		
Sulcus Superior (Pancoast)	9	*6*
Upper Lobe	95	*60*
Middle Lobe/Centrally located	30	*19*
Lower Lobe	23	*15*
**Induction Chemotherapy**		
Cisplatin/Etoposide	11	*7*
Cisplatin/Paclitaxel	146	*93*
**Concurrent Chemotherapy**		
Cisplatin/Etoposide	28	*18*
Cisplatin/Vinorelbine	129	*82*
**Radiotherapy Fractionation**		
conventional	77	*49*
accelerated-hyperfractionated	80	*51*
**Surgery**		
Segmentectomy	2	*1*
Lobectomy	105	*68*
Bi-Lobectomy	7	*4*
Pneumonectomy	40	*26*
Thoracotomy without resection	1	*1*
**Resection**		
microscopic complete (R0)	143	*92*
microscopic incomplete (R1)	9	*6*
macroscopic incomplete (R2)	4	*2*

### Sensitivity and specificity of histopathologic regression parameters to predict long-term survival

Sensitivity and specificity as well as the likelihood ratio were analyzed for the different histopathologic regression parameters to predict long-term survival. For the entire group of patients, pCR had the highest likelihood ratio among all histopathologic parameters that were significantly different from the value 1 for a useless test (Table 2). pCR and pCR-T (pathologic complete remission in the primary tumor in contrast to complete remission of tumor *and* mediastinal nodes) carry similar information and the vast majority (n = 41) of the 46 patients with pCR-T also achieved pCR. Decreasing the cut-off level for histopathologic tumor regression required for a positive histopathologic response from pCR over regression grades 2B to 2A (Reg-grade ≥2B to Reg-grade ≥2A) decreased the likelihood ratio for the association of the surrogate marker with long-term survival. Sensitivity of pCR as a predictor of survival was low (0.38), indicating that the majority of long-term survivors not achieving pCR following preoperative therapy were salvaged by tumor resection. MNC was found in approximately twice as many patients as pCR-T or pCR. This suggests a higher radiosensitivity of lymph node metastases as compared to the primary tumors. Sensitivity of MNC to predict survival was higher than that of pCR-T or pCR, but specificity was poor. The positive likelihood ratio for MNC was not significantly different from 1 (Table 2).

**Table 2:** **Sensitivity and specificity for the respective histopathologic response criterium to predict long term survival**

Patients with cN2-3 status						
	Number of patients	Long-term survivors	Sensitivity	Specificity	Sensitivity/(1-specificity)	CMH-test
all patients	157	72				
pCR	41	28	0.38 (0.27–0.50)	0.84 (0.77–0.92)	2.46 (1.39–4.38)	p = 0.0012
Reg-grade ≥ 2B	72	40	0.56 (0.44–0.67)	0.62 (0.52–0.73)	1.48 (1.05–2.08)	p = 0.025
Reg-grade ≥ 2A	135	66	0.92 (0.85–0.98)	0.19 (0.11–0.27)	1.13 (0.99–1.28)	p = 0.057
pCR-T	46	29	0.40 (0.29–0.51)	0.80 (0.71–0.88)	1.96 (1.18–3.25)	p = 0.008
MNC	85	44	0.61 (0.50–0.72)	0.52 (0.41–0.62)	1.26 (0.95–1.69)	p = 0.11

CMH measures the strength of association between the histopathologic response variable and long term survival which was defined as patient survival ≥ 36 months.

### Proportional hazard analysis of the clinical and pathological variables on survival

Proportional hazard regression containing all clinical and pathological variables found pCR, cN3, and gender as independent prognostic factors for survival using backward elimination of non-significant variables for reduced model selection at a significance level of α = 0.05 (model (1), Table 3).

**Table 3:** **Significant prognostic variables from proportional hazard regression analysis of survival data**

	model (1) containing clinical + pathol. covariates		standard model (2) containing clinical covariates alone	
variable	Hazard ratio	p-value	Hazard ratio	p-value
pCR	0.41 (0.25–0.67)	p = 0.0003	not included	
cN3	1.52 (1.01–2.29)	p = 0.047	1.59 (1.06–2.39)	p = 0.027
gender (female)	0.57 (0.38–0.87)	p = 0.008	0.63 (0.42–0.94)	p = 0.025
CT - tumor response	Backward eliminated	n.s.	0.59 (0.39–0.91)	p = 0.017

Model (1) contains all clinical and pathological covariates, as described under Methods. Backward elimination of non-significant variables was used for reduced model selection. CT-tumor response: partial or complete response after neoadjuvant radiochemotherapy in comparison to the pretreatment computed tomography imaging study.

The time era of treatment was not significantly associated with survival, indicating the absence of unrecognized period-dependent confounders.

With respect to the other histopathologic parameters, neither MNC without pCR nor Reg-grade ≥2B, or Reg-grade ≥2A without pCR carried important prognostic information in addition to pCR. After adjustment of the other prognostic factors, patients with MNC without pCR had a similar prognosis as patients without MNC and without pCR. 15 of 44 patients with MNC but without pCR were long-term survivors, 16 had known progression of disease (11 at distant and 2 at locoregional sites only, and 3 at both sites).

Response according to CT studies was not selected as an independent prognostic factor in addition to pCR.

No significant deviation from the proportional hazard assumption was found by analysis of the correlation between the Schoenfeld residuals and the rank order of failure times. Furthermore, time dependence of the hazard ratio did not become significant (p > 0.05).

In addition, the PHA parameter estimates from a standard model (model (2)) containing only pretreatment patient and tumor dependent clinical parameters with backward selection are shown in Table 3. Response according to CT studies became significant in the absence of histopathologic response parameters.

**Figure 2:**
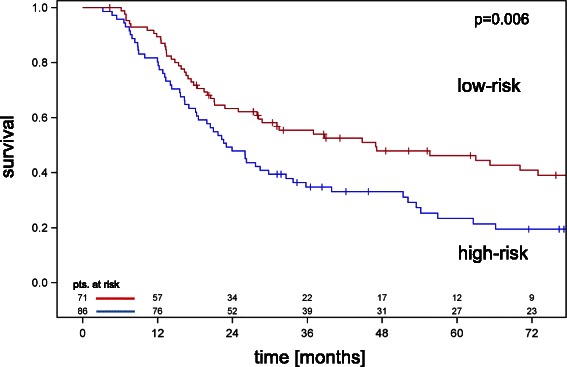
Cross-validated survival curves for patients classified as high and low risk including significant clinical and histopathologic parameters as well as age. The survival difference was significant using the log rank test (p = 0.006).

Both models were used to classify patients into equally sized high and low risk groups. A patient was assigned to the high risk group if the estimated hazard according to the model was larger than the median value in the sample of patients. To create continuous predictors, age was added as a continuous variable to the models. Older age was non-significantly associated with poorer survival by a hazard ratio of 1.02 per year increase. The model including histopathologic parameters led to a better separation of the survival curves between the high risk and low risk groups. The log-rank chi-square test statistic for the separation of the survival curves of the high and low risks groups was significantly larger using the model including pCR than the model including preoperative available parameters alone (p < 0.0001, chi^2^ test). Ten-fold cross-validation was performed in order to estimate the predictive value of both models on a data set independent of that one used for the model building process. The cross-validated Kaplan Meier curves for the high and low risk groups according to the model including histopathologic variables are shown in Figure 2. All 41 with pCR were sorted in the cross validated low risk group. A comparison of the log-rank statistics for the differences between the cross-validated Kaplan Meier curves for the high and low risk groups revealed a better separation by the model including histopathologic parameters than by the model including preoperative available parameters alone (p = 0.017, chi^2^ test).

In addition, we analyzed whether tumor response according to the CT-studies after neoadjuvant radiochemotherapy might predict pCR. The Spearman rank correlation between the response according to the CT-studies (PD/NC vs. PR/CR) and pCR was 0.21 (p < 0.05). None of the patients in this study, all of whom underwent surgery, had progressive disease after neoadjuvant radiochemotherapy. Among the 123 responding patients, there were 38 with pCR, while among the 34 non-responding patients only 3 were detected with pCR. The prognostic model (2) based on patient and tumor dependent pretreatment factors alone could also be improved by introduction of CT-response as an independent prognostic factor when histopathologic response parameters were not considered for prediction. The backward elimination procedure retained CT-response as a prognostic factor (hazard ratio 0.59 (0.39–0.91) for responding patients, p = 0.017, chi^2^ test).

PET/CT investigations before and after induction treatment were only available in less than half of the patients (n = 58). In an exploratory subgroup-analysis, deltaSUV_max_ in the primary tumor (= (SUV_max_ after induction chemotherapy)/(SUV_max_ before induction chemotherapy)) showed a positive correlation with pCR when using a cutoff-level of 0.3 separating responders (deltaSUV_max_ < 0.3) from non-responders (deltaSUV_max_ ≥ 0.3; Spearman rank correlation 0.28, p = 0.033). While 8 patients showed pCR in 17 responders, only 8 of 41 non-responders were found with pCR. When using CT-response criteria in this subgroup, 16 pCR patients out of 48 responders versus one pCR patient out of 10 non-responders were identified (Spearman rank correlation 0.19, p = 0.097). Due to the small number of patients, deltaSUV_max_ was not included in the prognostic model for survival.

**Figure 3:**
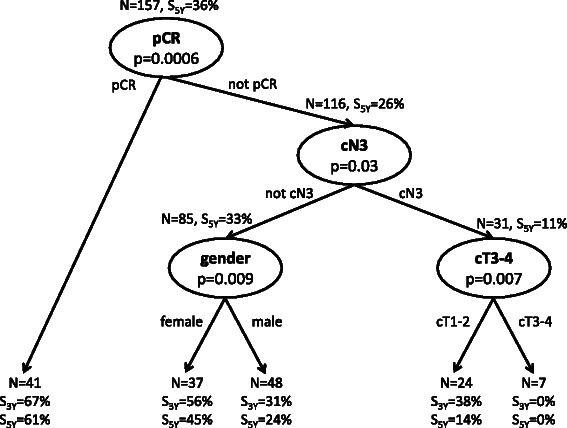
Regression tree diagram from recursive partition analysis using clinical and histopathologic prognostic parameters. For each node, the number of patients for the split, the log rank statistic for dissimilarity between the subgroups, and survival at 3 and 5 years (S_3y_, S_5y_) for the subgroups are given.

Recursive partitioning analysis (RPA) including all clinical and pathological predictor variables led to the regression tree shown in Figure 3. The top node contains pCR as the predictor variable. For patients without pCR, the cN3 lymph node category became important. For cN3 patients, high T category was selected as additional split to obtain prognostic subgroups.

Fourteen long term survivors were among the patients with cN3 disease. Five of them had a pCR. From the remaining 9 patients, all patients had cT2 tumors and seven showed a PR/CR to induction chemotherapy.

**Figure 4:**
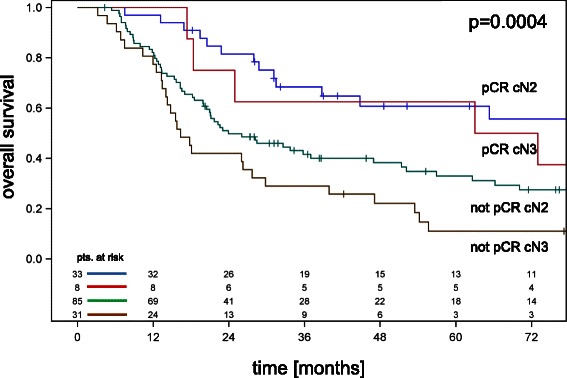
Kaplan Meier survival curves for the subgroups of patients with pathological complete remission (pCR), and without pCR (not-pCR) according to nodal category cN0-1, cN2, and cN3.

Kaplan Meier survival curves are given in Figure 4 for all patients with pCR and cN2, pCR and cN3, not-pCR and cN2 as well as not-pCR and cN3 following the splits according to the upper nodes of the regression tree in Figure 3. There was a significant effect of pCR on survival over the strata cN2 and cN3 (p = 0.0004, log rank test) and survival differed between cN2 and cN3 patients (p = 0.017, log rank test). Survival at 5 years was 61 ± 4% for patients with pCR and cN2, 63 ± 17% for pCR and cN3, and 33 ± 6% for not-pCR and cN2, as well as 11 ± 6% for not-pCR and cN3 patients, respectively.

### Proportional hazard analysis of the prognostic value of clinical and pathological characteristics on time to progression

Proportional hazard analysis revealed that complete remission by RECIST, and pCR were the sole prognostic parameters according to the proportional hazard model using backward selection that predicted time to progression (TTP). The hazard ratio associated with pCR on TTP was 0.34 (0.16–0.70).

## Discussion and conclusion

Important pre-treatment patient- and tumor-dependent prognostic factors found in this study were the clinical lymph node status, pneumonectomy, gender, adenocarcinoma histology, age, and Pancoast tumor localisation. All patients received induction chemotherapy, neoadjuvant radiochemotherapy und thoracotomy. These findings are in accordance with the Intergroup 0139 trial which also identified female sex as a favorable prognostic factor [2].

pCR was a strong prognostic factor for survival in this analysis and in other studies after neoadjuvant radiochemotherapy for locally advanced non-small cell lung cancer [9-11]. To substantiate the notion that pCR is associated with prolonged survival by improved long-term tumor control, the association between pCR and time to progression was analysed using PHA, and pCR was again observed as positive prognostic factor. The specificity for the prediction of long term survivors decreased substantially when the histopathologic criterium was broadened from pCR to MNC or regression grade ≥ 2B. Devitalisation of tumor in both the primary site and lymph nodes seems to be important to improve prognosis of patients treated with induction chemotherapy followed by radiochemotherapy in lung cancer. A similar observation was made in breast cancer after neoadjuvant chemotherapy. Disease-free survival was highest in breast cancer patients with pCR at the primary site and the involved lymph nodes [20]. The hazard ratio for disease-free survival in comparison to patients without histopathologic response increased from 0.45 for patients with no in situ residuals in the primary tumor and the lymph nodes to 0.52, 0.62, and 0.73, respectively, when patients with in situ residuals at the primary site, or patients with residually involved nodes but no invasive breast cancer at the primary site, or patients with focal invasive disease were included in the histopathologic response definition [21]. It was therefore concluded that ypTis, ypT1mic and ypN+ residuals alone are associated with an increased relapse risk and should not be considered in the definition of positive histopathologic response.

Downstaging to pCR after neoadjuvant radiochemotherapy can improve the prognosis for cN2 and cN3 patients and yields favorable long-term survival. In the cN2-3 group of our patients achieving pCR, five-year survival was 61%, well within the range of other studies [11]. The observation that pCR alone is a predictor of better prognosis is reflected by the position of pCR in the top node of the regression tree.

Selection of the optimal candidates for surgery remains a crucial point in daily clinical practice and is strongly influenced by current restaging capabilities [11,22]. Even considering the whole spectrum of procedures (from non-invasive radiological tools to more invasive surgical approaches), the restaging assessment often fails to predict the actual pathological response in this situation. Volume response to neoadjuvant radiochemotherapy from sequential CT-studies has been found to be correlated with pCR and prognosis after neoadjuvant radiochemotherapy by this study and is an important preoperative prognostic factor that, however has not the predictive power of pCR itself. FDG-PET/CT has been investigated in this situation adding at least some valuable information on treatment response in these patient cohorts [23,24]. When using cut-off levels of deltaSUV_max_ around 0.3 to define responders a positive correlation with pCR was observed. Whether PET-response to neoadjuvant radiochemotherapy is a better prognostic factor for overall survival than CT-response remains an open question.

The present study is a retrospective investigation of a group of consecutive patients treated at a single institution. Although the physicians’ team has remained constant through the long duration of patients inclusion some changes of the induction as well as the concurrent treatment have taken place. Thus, some intrinsic bias cannot be excluded entirely and when translating our findings into a general setting of preoperative treatment this should be taken into account.

pCR improved the survival risk discrimination between equally sized low risk and high risk groups using a clinico-pathologic prognostic model according to cross-validated Kaplan-Meier curves. There is a considerable interest to refine prognostic models for lung cancer by gene expression signatures and whole genome sequencing [25,26]. Given the dominant prognostic significance of pCR after neoadjuvant radiochemotherapy using cisplatin containing doublets, pCR should be included as a prognostic factor in studies of patients after neoadjuvant radiochemotherapy. If further treatments are considered after neoadjuvant radiochemotherapy and resection, as in the Intergroup 0139 or the RTOG 02–29 trial [2,6], the prognostic model including pCR might help to identify patients who benefit most from additional postoperative treatment or treatment intensification, respectively.

## Abbreviations

CMH: Cochrane-Mantel-Haenszel
CR: Complete remission
CT: Computed tomography
CTC: Common toxicity criteria
MNC: Mediastinal nodal clearance
NC: No change
NOS: Not otherwise specified
NSCLC: Non-small cell lung cancer
OS: Overall survival
pCR: Pathologic complete remission
pCR-T: Pathologic complete remission in the primary tumor
PD: Progressive disease
PET/CT: Positron emission tomography/computed tomography
PHA: Proportional hazard analysis
PR: Partial remission
Reg-grade: Grade of remission
RPA: Recursive partitioning analysis
SUV_max_: Maximum standardized uptake value
TTP: Time to progression
